# SCAMP1 silencing inhibits proliferation by attenuating multiple pro-survival signaling pathways in gastric cancer

**DOI:** 10.7150/jca.99610

**Published:** 2024-09-09

**Authors:** Gang Ma, Yang Yang, Fenglin Cai, Bin Ke, Jingyu Deng

**Affiliations:** 1 Department of Gastric Surgery, Tianjin Medical University Cancer Institute & Hospital, National Clinical Research Center for Cancer; Tianjin Key Laboratory of Digestive Cancer, Tianjin; Tianjin's Clinical Research Center for Cancer, Tianjin, 300060, P. R. China.; 2 Department of Anesthesiology, Tianjin Medical University Cancer Institute & Hospital, National Clinical Research Center for Cancer; Tianjin Key Laboratory of Digestive Cancer, Tianjin; Tianjin's Clinical Research Center for Cancer, Tianjin, 300060, P. R. China.

**Keywords:** SCAMP1, Proliferation, Endocytosis, Gastric Cancer

## Abstract

Secretory carrier-associated membrane protein 1 (SCAMP1) is the most universally expressed member of the SCAMP family, and its ability to facilitate endocytosis was demonstrated approximately two decades ago. Nevertheless, its roles in cancer biology are largely unknown, although its expression is significantly increased in most cancer types. Herein, we examined the expression of SCAMP1 in gastric cancer (GC) tissues and found that it was aberrantly increased and positively correlated with tumor size and lymph node metastasis. More importantly, increased SCAMP1 expression was associated with poor prognosis in patients with GC. Functional experiments demonstrated that SCAMP1 knockdown markedly suppressed the proliferation of GC cells *in vitro* and *in vivo*. RNA sequencing assays demonstrated that SCAMP1 knockdown altered the expression profile of GC cells, and a significant portion of the altered genes were enriched in receptor tyrosine kinases and their related downstream signaling pathways. Immunoblotting confirmed that the Akt/MAPK/Stat signaling pathway was strongly attenuated in GC cells with SCAMP1 depletion. Taken together, these results demonstrated that SCAMP1 drives hyperproliferation in GC cells, thus suggesting that further investigation into the mechanisms and translational value of SCAMP1 in treating patients with GC is warranted.

## Introduction

Gastric cancer (GC) remains a global health problem, as it was the fifth most common cancer and the fifth leading cause of cancer-related deaths in 2022 1, 2. In terms of the classification of GC, the most commonly used Lauren criteria, which are based on histologic features, divide GC tissues into two major subtypes: intestinal and diffuse 3. Intestinal GC is strongly associated with *Helicobacter pylori* infections and other environmental factors, whereas diffuse GC is usually attributable to genetic factors, such as mutations in the *CDH1* and *RHOA* genes 3. Recent technological advancements have prompted the molecular classification of GC, as evidenced by The Cancer Genome Atlas (TCGA), which categorizes GC into the Epstein-Barr virus-positive, microsatellite unstable, genomically stable, and chromosomally unstable subtypes 3. Intestinal GC usually displays sequential carcinogenesis events, including chronic gastritis, intestinal metaplasia and dysplasia; conversely, early-stage diffuse GC usually contains signet ring cells (SRCs) 3. As diffuse GC progresses, malignant cells are more inclined to metastasize to distal organs 3. Surgical treatment is still the primary therapeutic option for most patients with GC. Notably, neoadjuvant therapeutic strategies combining conventional platinum/fluoride-based drugs, targeted drugs (such as trastuzumab) or immune checkpoint blockade agents (such as nivolumab) can greatly improve survival in a subset of patients with GC 3. However, the high heterogeneity of GC and the advanced stage at first diagnosis limit its therapeutic efficacy and result in unfavorable outcomes for patients with GC. Thus, it is vital to identify the underlying mechanisms of the development and progression of GC.

Endocytosis is an intricate process by which cells deliver surface lipids and proteins, as well as extracellular materials, into cells 4. Multiple pathways of endocytosis, such as the best-studied clathrin-mediated endocytosis pathway, have been identified in mammalian cells, and each route is responsible for the internalization of distinct types of surface and extracellular cargos 4. Dysfunctional endocytosis, which is the primary regulator of crosstalk between cells and their environment, contributes to tumor initiation, progression, and metastasis 4, 5. More intriguingly, due to the fact that the internalization of tumoricidal antibodies targeting surface proteins of cancer cells, such as antibody‒drug conjugates (ADCs), relies on endocytosis, the practice of manipulating endocytosis to improve drug efficacy has become a popular research topic in recent years 6. Hundreds of proteins orchestrate proper and effective endocytosis, among which the secretory carrier-associated membrane protein (SCAMP) family, which consists of five members (SCAMP1/2/3/4/5), is implicated in vesicle transport pathways between the plasma membrane and internal recycling network 7, 8, 9. As the most abundantly expressed and widely distributed member of this family, SCAMP1 reportedly affects the stability of fusion pores during endocytosis after their formation 10. Interestingly, SCAMP1 and SCAMP3 can be phosphorylated by activated epidermal growth factor receptor (EGFR) at distinct tyrosine residues, and phosphorylated SCAMPs then bind to EGFR to facilitate the internalization and degradation of this receptor 11. This finding suggested that SCAMPs likely play roles in cancer biology and may be promising intervention targets for treating cancer. However, fairly little is known about the functions of SCAMPs, including SCAMP1, in cancer.

Due to the fact that there are few studies about SCAMP1 in cancer biology, we attempted to investigate the function and molecular mechanisms of this protein in GC cells, especially because we observed that *SCAMP1* expression was aberrantly increased across most cancer types, including GC, according to TCGA data. This study provided more knowledge on the role of SCAMP1 in the proliferation of GC cells.

## Materials and methods

### Patient specimens

GC and adjacent normal tissue samples were collected from 127 patients in total who underwent curative gastrectomy at Tianjin Medical University Cancer Hospital (Tianjin, China) between January 2004 and September 2007. All of these eligible patients were suffered from histologically confirmed and locally advanced adenocarcinoma of the stomach, and patients suffered with other subtypes of GC, such as gastric squamous cell carcinoma, were excluded from this study. In addition, patients receiving any kind of therapies, including chemotherapy or radiotherapy, before the operation were excluded from this study. Among these enrolled patients, under-65 age group included 81 patients, while over-65 age group contained 46 patients. Moreover, 87 participants were men patients and the other 40 were women patients. Follow-up was conducted every 3-6 months and was completed in September 2012. The median time was 32.0 months (range: 3-72 months). Written informed consent was obtained from each patient.

### TCGA dataset analysis

Pancancer analysis of SCAMP1 mRNA levels was performed via online bioinformatic tools (https://www.xiantao.love/). The correlation between SCAMP1 mRNA levels and survival in patients with GC from The Cancer Genome Atlas (TCGA) was assessed by using the R package “Survminer”.

### Cell culture

The human GC cell lines NCI-N87, SNU-1, KATO III, AGS, and SNU-16 were purchased from American Type Culture Collection (ATCC, USA). The human GC cell line HGC-27 and the human immortalized gastric epithelial cell line GES-1 were obtained from the National Infrastructure of Cell Line Resource (Beijing, China). The human GC cell line MKN45 was kindly provided by Prof. Hui Li from the Department of Gastrointestinal Cancer Biology at Tianjin Medical University Cancer Institute and Hospital (Tianjin, China). The HEK293T cell line was a generous gift from Prof. Zhihua Liu from the National Cancer Center/Cancer Hospital (Beijing, China). RPMI-1640 supplemented with 10% fetal bovine serum (FBS; Newzerum, Christchurch, New Zealand) was used as the routine culture medium for the NCI-N87, SNU-1, SNU-16, HGC-27, and MKN45 cells. AGS cells were cultured in F12K medium supplemented with 10% FBS. KATO III cells were maintained in IMDM supplemented with 20% FBS. HEK293T cells were cultured in DMEM supplemented with 10% FBS. All of the culture media were supplemented with 1% penicillin/streptomycin. All of the cells were maintained in a cell incubator with 5% CO_2_ at 37 °C. The duration of culture did not exceed two months. All of the cell lines were verified to be *Mycoplasma*-negative by using the PCR Mycoplasma Detection Set (TaKaRa).

### Reagents and antibodies

Puromycin and Cell Counting Kit-8 (CCK-8) were purchased from MedChemExpress (NJ, USA). The Click-iT™ EdU Alexa Fluor™ 488 kit (C10425) and Pierce™ BCA Protein Assay Kit were purchased from Thermo Scientific (USA). RNAiso plus and TB Green Premix Ex TaqTM II (RR820A) were obtained from TaKaRa (Shiga, Japan). A GoScript™ Reverse Transcription Kit (A5000) was purchased from Promega (WI, USA). The phosphatase inhibitor PhosSTOP™ and the protease inhibitor cOmplete™ Protease Inhibitor Cocktail were obtained from Merck (Rahway, NJ, USA).

Primary antibodies against Akt (C67E7), phospho-Akt (Ser473) (193H12), ERK1/2 (137F5), phospho-ERK1/2 (Thr202/Tyr204) (197G2), STAT3 (124H6), phospho-STAT3 (Tyr705) (3E2), and vinculin (E1E9V) were purchased from Cell Signaling Technology (Danvers, MA, USA). The primary antibody against SCAMP1 (15327-1-AP) was obtained from Proteintech (Rosemont, IL, USA).

### Lentivirus and establishment of stable cell populations

To stably reduce the endogenous expression of SCAMP1, two target shRNAs were subcloned and inserted into the lentiviral vector pSIH-H1-puro, which was generously provided by Prof. Zhihua Liu from the National Cancer Center/Cancer Hospital, Beijing, China. Lentivirus was generated as previously described 12. One shRNA targeting SCAMP1 (shS1#1) targeted the sequence 5'-CCAAACCTGTAGTTACAGAAA-3', and another (shS1#2) targeted the sequence 5'-CCTCAGTCAACATGGTAGAAA-3'. The empty pSIH-H1-puro vector was used as the negative control (shCtrl). Puromycin (2 μg/ml) was used to construct stable cell populations.

### RNA extraction and quantitative real-time polymerase chain reaction

The cultured cells were harvested when they were approximately 80% confluent, and total RNA was obtained by using RNAiso Plus (TaKaRa) according to the manufacturer's instructions. After the concentrations of the RNAs were determined via NanoDrop™ One (Thermo Fisher Scientific), potentially contaminated DNAs were removed from the extracted RNAs by adding 1 unit of DNase I (New England Biolabs) to 1 μg of RNA and incubating for 30 min at 37 °C. The processed RNAs with an A260/A280 ratio of approximately 2.0-2.1 were reverse-transcribed into complementary DNA (cDNA) by using a GoScript™ Reverse Transcription Kit; subsequently, quantitative real-time polymerase chain reaction was performed as previously described 12. The primers for SCAMP1 were as follows: forward, 5'-GAAACCAACAGAGGAACATCCAG-3'; reverse, 5'-CCGACGATCTAATTCTGCGGCT-3'. GAPDH was used as the internal control to normalize gene expression levels, and the utilized primers were as follows: forward, 5'-TGCACCACCAACTGCTTAGC-3'; reverse, 5'-GGCATGGACTGTGGTCATGAG-3'. The mRNA expression of SCAMP1 was calculated by using the comparative 2^-ΔΔCt^ method.

### Immunoblotting

The cells that were used for immunoblotting assays were harvested when they grew to approximately 80% confluence. The proteins were extracted by using cell lysis buffer (20 mM Tris, 0.15 M NaCl, 0.05 M EDTA, 1% NP-40, pH=7.5) supplemented with PhosSTOP™ and cOmplete™ Protease Inhibitor Cocktail. The protein concentrations were determined by using the BCA method, and 15 μg of the extracted proteins was then subjected to SDS‒PAGE. Immunoblotting assays were conducted as previously described 12. Vinculin was used as the loading control. The dilutions of the primary antibodies against SCAMP1 or vinculin were 1:1000.

### Immunohistochemistry (IHC)

IHC was conducted, and the stain scores were measured as previously described 12. Briefly, the dilution of the primary antibody against SCAMP1 (Proteintech, 15327-1-AP) was 1:200. The stain scores were calculated by multiplying the stain intensities and the scores of the positively stained proportion. The staining intensities were determined as follows: 0, negative; 1, weak; 2, moderate; and 3, strong. The scores of the positively stained cell proportion were as follows: 0, not positive; 1, <10%; 2, 10-35%; 3, 35-75%; and 4, >75%. Thus, possible staining scores of 0, 1, 2, 3, 4, 6, 8, 9, and 12 were obtained for SCAMP1 protein expression in GC and adjacent normal tissues. The cutoff value between high and low expression of SCAMP1 was set as 6, namely 0-4 were considered low while 6-12 high.

### Cell proliferation assays

*In vitro* cell proliferation was evaluated via CCK-8 and EdU incorporation assays. These assays were performed according to the manufacturers' instructions. Additionally, twelve five-week-old female Balb/c nude mice in total were purchased from GemPharmatech (Jiangsu, China) and housed in an animal facility located at Tianjin Medical University Cancer Institute and Hospital. These mice were randomly into shCtrl/shS1#1 and shCtrl/shS1#2 group and each group had six mice. Three mice were housed in each individually ventilated cage on a 12-h/12-h light/dark cycle with food and water available *ad libitum*. The living status of the mice was checked once a day throughout this experiment. After these mice were housed for 7 days, approximately 2 × 10^6^ control (shCtrl) and SCAMP1-depleted (shS1#1 or shS1#2) NCI-N87 cells suspended in 100 μl of PBS were subcutaneously injected into the left or the right dorsal flank of the mice, respectively. The tumor masses were measured every four days. Tumor volumes were calculated by using the following formula: V = (width^2^ × length) × 0.5. The diameter of the tumor mass was greater than 1.5 cm in mice was defined as the endpoint of the experiment. The mice were sacrificed 23 days after the subcutaneously implantation of the cells. All of the animal subjects survived until the end of the experiment without signs of illness. The mice were euthanized by using an overdose of carbon dioxide (CO₂) at a displacement rate of 30% volume per minute. The deaths of these mice were verified by a combination of cessation of heartbeat and respiration, as well as loss of corneal and toe pinch reflexes, before the tumors were harvested and weighed. All efforts were made to minimize the number of mice that were used and their suffering throughout the experiment.

### RNA sequencing assay

Total RNA was isolated from shCtrl and shS1#1 NCI-N87 cells by using TRIzol™ Reagent (Thermo Fisher Scientific). The sequence and data analysis, including Gene Ontology (GO) and Kyoto Encyclopedia of Genes and Genomes (KEGG) pathway enrichment, were conducted by Novogene (Beijing, China). Genes with |log2FC| > 1 and *P* < 0.05 were defined as being differentially expressed genes (DEGs). The raw data were deposited under GSE252526 in the GEO database.

### Statistical analysis

All of the experiments, except for the animal, IHC, and RNA-seq assays, were independently repeated three times. The presented data are expressed as the mean ± standard deviation (SD). Paired* t-*test was used to determine significant differences between two groups. One-way ANOVA with Dunnett's *post hoc* test or two-way ANOVA with Bonferroni *post hoc* test, which are indicated in the figure legends, was conducted to determine statistical significance among at least three groups. The chi-square test was used to evaluate the correlation between SCAMP1 expression and clinicopathological properties. Overall survival (OS) was analyzed via the Kaplan-Meier method, and the log-rank test was used to measure statistical significance. The multivariate analysis of OS was conducted by using the *Cox* proportional hazard model with forward-step procedures. GraphPad Prism (version 8.0) was used to calculate all of the significant differences and to draw relative charts. A *P* value < 0.05 was considered to indicate statistical significance. *, **, ***, and **** indicate *P* < 0.05, *P* < 0.01, *P* < 0.001, and *P* < 0.0001, respectively.

## 3. Results

### 3.1 SCAMP1 is highly expressed in GC tissues and indicates unfavorable patient outcomes

**Figure 1:**
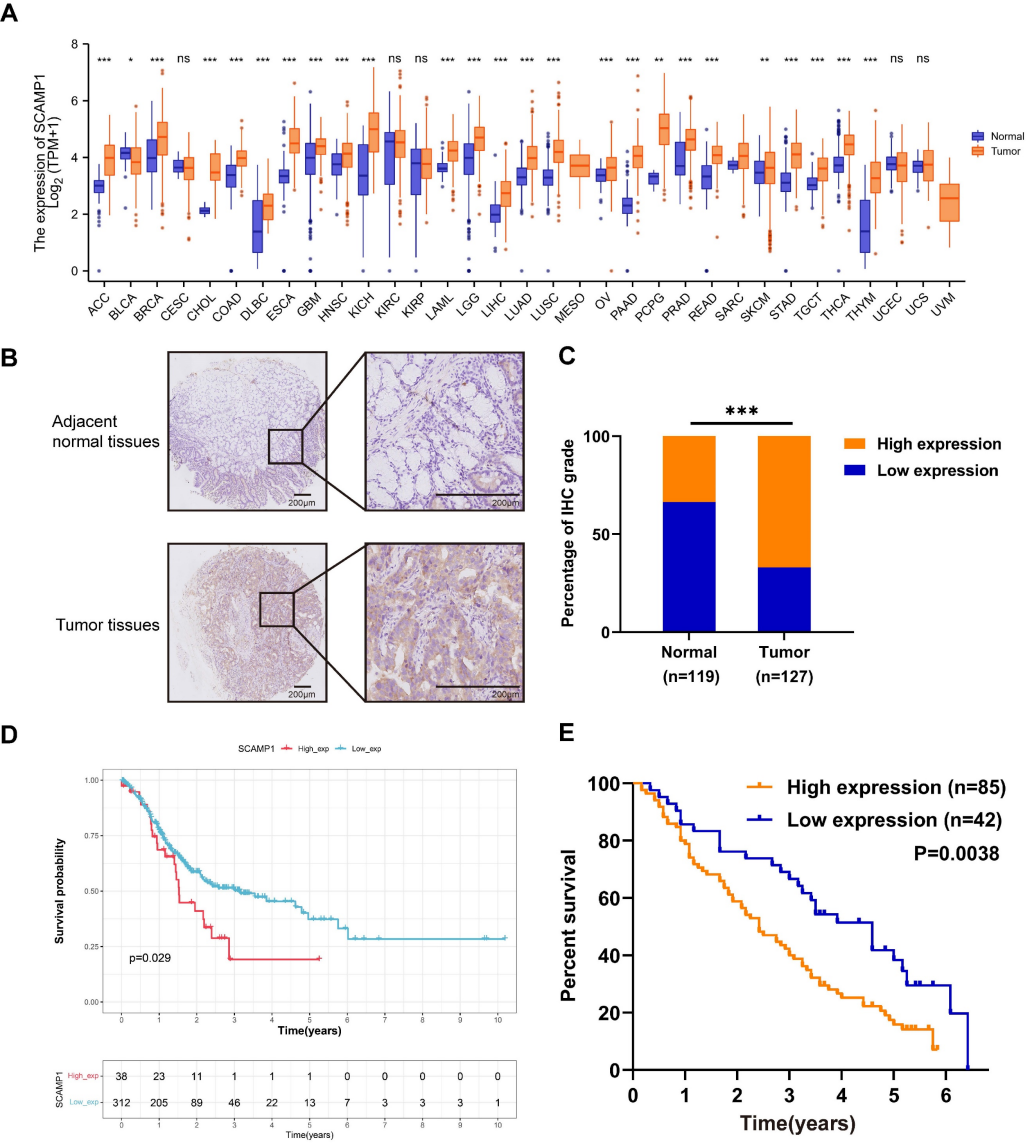
SCAMP1 expression is upregulated in GC tissues and associated with unfavorable overall survival in patients with GC. (**A**) Mining of TCGA datasets demonstrated that SCAMP1 expression is markedly increased in most cancer types, including GC (Ca: 375, Normal: 391). (**B**) Representative images of IHC staining for SCAMP1 in GC and adjacent normal tissues. Scale bars, 200 μm. (**C**) The IHC scores of SCAMP1 expression in our institutional GC and normal tissue samples. The cutoff value between high and low expression of SCAMP1 was set as 6. (**D and E**) A high SCAMP1 level indicates poor prognosis in patients with GC in the TCGA cohort (*P* = 0.029) (**D**) and in patients with GC enrolled at the Tianjin Medical University Cancer Institute and Hospital (*P* = 0.038) (**E**). ***, *P* < 0.001.

We first examined *SCAMP1* expression across various cancer types from the TCGA. We found that *SCAMP1* mRNA expression was markedly increased in most of the tested cancer types, including stomach adenocarcinoma (STAD) tissues, compared with normal tissues (*P* < 0.001). These findings strongly suggested that SCAMP1 likely plays critical roles in GC cells (Figure 1A). Afterwards, SCAMP1 protein levels were tested via IHC in in-house GC and matched normal tissue samples. IHC demonstrated that SCAMP1 was mainly positively stained in the cytoplasm, and the staining intensity of SCAMP1 was significantly greater in GC tissue samples than in adjacent normal tissue samples (Figure 1B). Specifically, 85 out of 127 GC samples strongly expressed SCAMP1, whereas 40 of 119 normal tissues strongly expressed SCAMP1 (*P* < 0.001) (Figure 1C, Table 1). Moreover, increased SCAMP1 protein levels were associated with increased tumor size (*P* = 0.028) and positive lymph node metastasis (*P* = 0.015) (Table 2). A previous study showed that high SCAMP1 expression was indicative of poor prognosis in patients with pancreatic adenocarcinoma 13, which prompted us to perform a prognostic analysis of SCAMP1 in patients with GC. Analysis of GC patients from TCGA demonstrated that patients with higher SCAMP1 levels had shorter survival (OS cutoff point: high, 28.8 months vs. low, 68.4 months; *P* = 0.029) (Figure 1D). Consistently, prognostic analysis based on our institutional GC tissues further demonstrated that increased SCAMP1 expression was positively correlated with worse survival (median OS: high, 29 months vs. low, 55 months; *P* = 0.0038) (Figure 1E). More importantly, multivariate *Cox* regression analysis demonstrated that elevated SCAMP1 levels were an independent prognostic factor for worse survival in patients (*P* = 0.036) (Table 3). Taken together, these results suggest that SCAMP1 likely plays vital roles in GC cells.

### 3.2 SCAMP1 knockdown inhibits the proliferation of GC cells *in vitro* and *in vivo*

**Figure 2:**
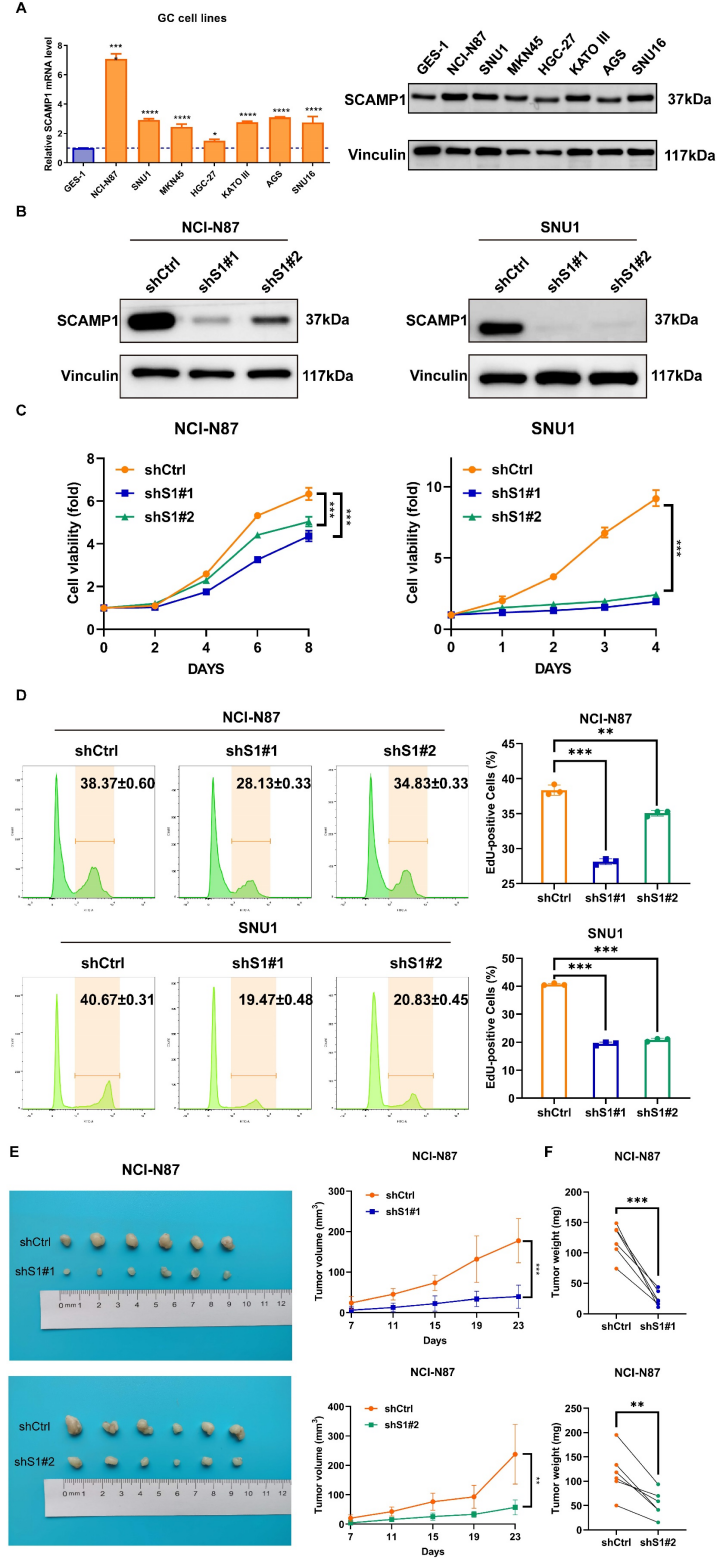
SCAMP1 knockdown markedly attenuated the proliferation of GC cells *in vitro* and *in vivo*. (**A**) The mRNA and protein levels of endogenous SCAMP1 in GES-1 cells and a panel of GC cell lines. The difference in SCAMP1 mRNA expression between GES-1 cells and each GC cell line was evaluated by using one-way ANOVA with Dunnett's *post hoc* test, which demonstrated that SCAMP1 mRNA levels in all GC cell lines were significantly increased relative to that in GES-1 cells (*, *P* < 0.05; ****, *P* < 0.0001). (**B**) Immunoblotting was used to validate the efficacy of endogenous SCAMP1 knockdown in NCI-N87 (left) and SNU-1 (right) cells. (**C**) CCK-8 assays demonstrated that SCAMP1 depletion reduced the viability of NCI-N87 (left) and SNU-1 (right) cells. Statistical significance was determined using one-way ANOVA with Dunnett's* post hoc* test. (**D**) EdU incorporation assays showed that the proportions of replicating SCAMP1-depleted NCI-N87 (left) and SNU-1 (right) cells decreased markedly. One-way ANOVA with Dunnett's* post hoc* test was used to assess statistical significance. (**E** and** F**) SCAMP1 knockdown diminished the proliferation of NCI-N87 cells *in vivo*, as demonstrated by reduced tumor volumes (n = 6) (**E**) and decreased tumor weights (n = 6) (**F**). Paired *t*-test was used to evaluate statistical significance in E and F. **, *P* < 0.01; ***, *P* < 0.001.

As highly expressed SCAMP1 is correlated with the hyperproliferative and invasive phenotypes of GC cells (Table 2), we explored the function of this protein in proliferation because the role of SCAMP1 in proliferation is largely unknown, whereas its role in the invasion of malignant cells has been described in previous reports 14, 15. We examined the expression of SCAMP1 in GES-1 cells and a panel of GC cell lines and found that compared with GES-1 cells, most of the GC cell lines displayed markedly greater SCAMP1 expression at both the mRNA and protein levels (Figure 2A). Endogenous SCAMP1 expression in NCI-N87 and SNU-1 cells was reduced with shRNAs against SCAMP1 mRNA (Figure 2B), and the proliferative capacity of the two cell populations with depleted SCAMP1 was subsequently analyzed. CCK-8 assays showed that SCAMP1 depletion markedly attenuated viability in both NCI-N87 and SNU-1 cells within the indicated time intervals (Figure 2C). In addition, an EdU incorporation assay confirmed that SCAMP1 knockdown dramatically decreased the proportion of the replicating GC cell population in NCI-N87 and SNU-1 cells (Figure 2D). We further assessed the effect of SCAMP1 on the proliferation of GC cells *in vivo* by subcutaneously injecting SCAMP1-depleted NCI-N87 cells and control cells into BALB/c nude mice. NCI-N87 cells with SCAMP1 knockdown grew more slowly than control cells, and SCAMP1 deficiency limited the weights of the harvested tumor masses derived from NCI-N87 cells (Figure 2E and 2F). Collectively, these data support the idea that SCAMP1 is essential for sustaining proliferation in GC cells.

### 3.3 SCAMP1 knockdown blunts critical pro-survival signaling pathways

**Figure 3:**
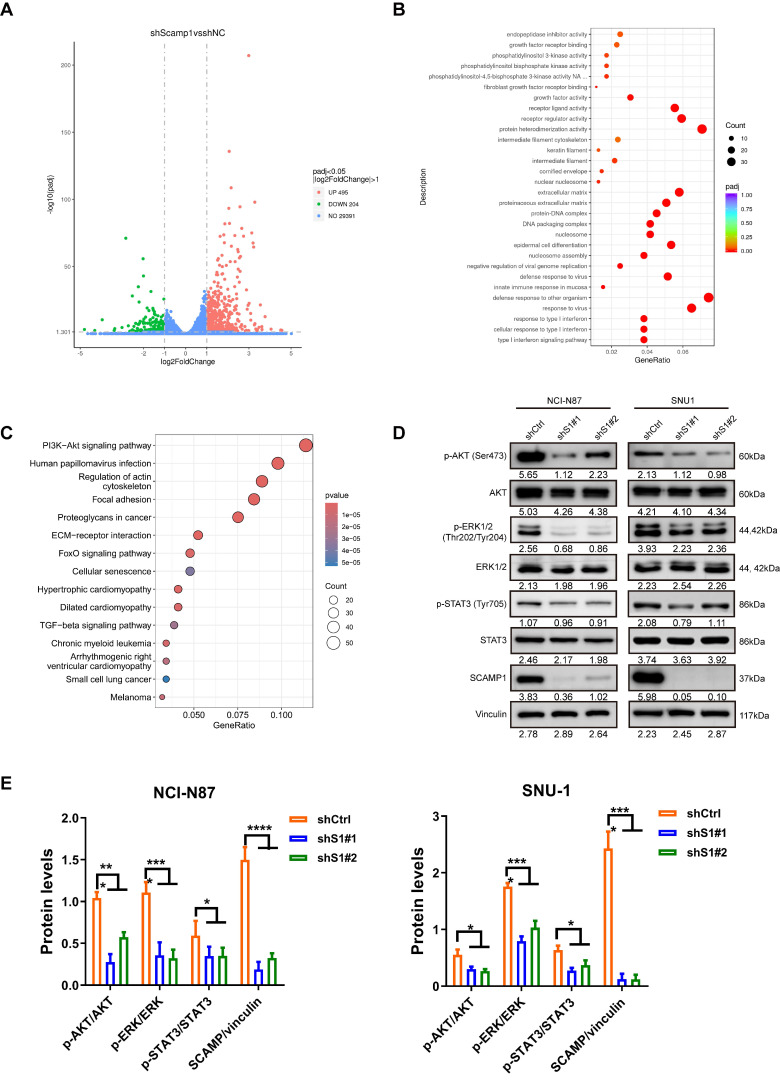
Depletion of SCAMP1 deactivated multiple cancer-promoting signaling pathways. (**A**) RNA-seq demonstrated that the expression of 495 genes increased while the expression of 204 genes decreased in shS1#1 NCI-N87 cells compared with shCtrl NCI-N87 cells. (**B**) GO analysis showed that the differentially expressed genes (DEGs) were primarily enriched in membrane receptors and their implicated signaling pathways. (**C**) The GC cohort in TCGA was stratified into low-SCAMP1-expression and high-SCAMP1-expression groups, and the DEGs between these two groups were also enriched in signaling pathways similar to those revealed by RNA-seq. (**D**) Immunoblotting assays showing that SCAMP1 knockdown markedly reduced the levels of p-Akt (Ser473), p-ERK1/2 (Thr202/Tyr204), and p-STAT3 (Tyr705). (**E**) The ratios of p-AKT/AKT, p-ERK/ERK, p-STAT3/STAT3, and SCAMP/vinculin remarkably decreased in shS1#1/#2 cells relative to those in shCtrl cells. Two-way ANOVA with Bonferroni *post hoc* test was conducted to evaluate the statistical significance. *, *P* < 0.05; ***, *P* < 0.001; ****, *P* < 0.0001.

To understand how SCAMP1 depletion abrogates propagation in GC cells, RNA sequencing analysis of control and SCAMP1-depleted NCI-N87 cells was performed, and the results demonstrated that the expression of 699 genes changed by more than twofold, among which 495 genes exhibited increased expression, whereas 204 genes exhibited reduced expression (Figure 3A). Gene Ontology (GO) analysis indicated that a significant portion of these DEGs were enriched in ligand‒receptor interactions, such as fibroblast growth factor receptor binding, and downstream signaling pathways, such as the PI3K-Akt pathway (Figure 3B). We also examined signaling pathways affected by increased SCAMP1 expression by dividing GC samples deposited in the TCGA database into SCAMP1-high-expression and SCAMP1-low-expression groups. Similarly, the ECM-receptor interaction and the PI3K-Akt signaling pathway were also highly detected (Figure 3C). These results strongly suggested that SCAMP1 likely regulates signal transduction via membrane-anchored receptors. Moreover, SCAMP1 knockdown decreased the levels of phosphorylated AKT (Ser473), ERK1/2 (Thr202/Tyr204) and STAT3 (Tyr705) (Figure 3D), which are primary downstream protumoral effectors of kinase-coupled membrane receptors, such as fibroblast growth factor receptor (FGFR) 16. Meanwhile, the ratios of p-AKT/AKT, p-ERK/ERK, p-STAT3/STAT3, and SCAMP/vinculin were further analyzed, demonstrating that all of them remarkably decreased in shS1#1/#2 cells compared to those in shCtrl cells (Figure 3E). These results demonstrate that SCAMP1 promotes proliferation by activating multiple oncogenic signaling pathways.

## 4. Discussion

Endocytosis profoundly affects communication between the external environment and cells; thus, dysfunctional endocytosis pathways are unsurprisingly implicated in almost all hallmarks of cancer cells, such as hyperproliferation, metastasis, and immune evasion 4, 5. More importantly, recent publications have highlighted the pivotal role of endocytosis in controlling the internalization of drugs into tumor cells; therefore, this process is emerging as a potentially valuable intervention target to improve therapeutic efficacy 4. Prochlorperazine, which is an antipsychotic drug, was shown to be capable of reversibly inhibiting the inward trafficking of membrane proteins, thus potentiating the NK cell-mediated antibody-dependent cellular cytotoxicity (ADCC) of the tested antibodies, including trastuzumab, cetuximab and avelumab, in nonresponsive and resistant human tumors 17. In addition, multiple chemical inhibitors have been reported to block different pathways of endocytosis, and some of them have shown anti-tumoral effects 4. However, these antineoplastic effects can vary depending on the treated tumor type. Dynasore, which is a potent inhibitor of endocytosis, was found to be more effective in hematological tumor cells than in solid cancer cells 18. Dysfunctional endocytic proteins are distinct across tumor types. In this study, we demonstrated that SCAMP1 expression was aberrantly increased in GC tissues and was positively correlated with tumor size and lymph node metastasis. More intriguingly, functional analysis demonstrated that SCAMP1 knockdown markedly impaired the proliferation of GC cells. Taken together, these results strongly suggest that SCAMP1 contributes greatly to the hyperproliferative phenotype. Unfortunately, there are no commercially available chemical inhibitors of SCAMP1 to date; however, specific inhibitors of SCAMP1 and their potential translational value in GC merit further identification.

Receptor tyrosine kinases (RTKs) are commonly hyperactivated to promote aggressive tumor phenotypes. The endocytosis machinery plays key roles in controlling the recycling of RTKs and their downstream signaling pathways. For example, EGFR has been shown to be highly expressed in GC, and the recycling of this receptor markedly drives the survival of GC cells 19. Mechanistically, vacuolar protein sorting-associated protein 35 (VPS35) is responsible for the endosomal transportation of EGFR in GC cells, and the inhibition of VPS35 expression enhances sensitivity to EGFR inhibitors, such as erlotinib 19. Similarly, SCAMP1 knockdown significantly influenced RTK-mediated signaling pathways, such as the PI3K-Akt and MAPK signaling pathways, as confirmed via RNA-seq analysis, TCGA GC sample data mining, and immunoblotting assays. These preliminary data indicate that depletion of SCAMP1 likely disturbs the proper function of RTKs in GC cells. Of note, the surface proteome analysis demonstrated that the most differentially expressed proteins between sh-NC and sh-SCAMP1 cells are enriched in oxidative phosphorylation and purine metabolism (Data not shown). Therefore, the investigations are under progress to unravel the intricate interactions between SCAMP1 and these pro-tumoral signaling pathways in GC cells.

Notably, it is becoming increasingly evident that advanced tumors not only remodel their microenvironment but also markedly disturb systematic homeostasis via the neuroendocrine system 20. Several studies have revealed a potential correlation between SCAMP1 and neurological system function. SCAMP1 was shown to be enriched in synaptic vesicles 21, thus suggesting that the endocytosis controlled by this protein is likely involved in neurotransmitter delivery. Recent studies have demonstrated that neural SCAMP1 is involved in regulating various behaviors. In Drosophila, simultaneous genetic depletion of SCAMP1-SCAMP5 in neurons elicited abnormal behaviors, such as climbing difficulties 22. Presynaptic machinery containing vesicular glutamate transporter 3 (VGluT3) is vital for the transmission of auditory information to inner hair cells (IHCs) in the brain 23. Further proteomic analysis demonstrated that SCAMP1 was enriched in VGluT3-positive membrane vehicles after hearing onset in mice 23, thus indicating that this protein likely plays a role in hearing onset. Unfortunately, the evidence to support that tumor-derived SCAMP1 systematically influences malignant progression is sparse; however, the emerging functions of SCAMP1 in synaptic transmission will prompt further investigations into its possible roles in broader areas.

Notably, there are a few limitations in the present data. First, whereas disease-free survival (DFS) analysis is vital for evaluating the clinical significance of SCAMP1, we cannot provide the analytic results since we did not have the relevant data of the patients enrolled in this study. Secondly, the tissues used in this study were collected 20 years ago, and some useful clinicopathological data, such as DFS data, are lack. Although the collection of patients' tissue samples is in progress recently, the emergence of neoadjuvant chemotherapy and immunotherapy greatly reduces the number of the patients who meet the criteria. However, more mechanistic details of SCAMP1 and its correlation with GC progression will be further investigated in our lab.

In summary, we demonstrated that SCAMP1 knockdown suppresses proliferation by deactivating multiple tumor-promoting signaling pathways in GC cells. These initial results further suggest that SCAMP1 likely orchestrates the recycling and function of specific membrane proteins, which may prompt further explorations of its molecular mechanisms and translational potential in GC treatment.

## Figures and Tables

**Table 1:** SCAMP1 expression in GC and adjacent normal tissues

	SCAMP1		χ^2^	*P* value
	Low	High		
Normal tissues	79(66.39%)	40(33.61%)	27.283	<0.001
Tumor tissues	42(33.07%)	85(66.93%)		

**Table 2:** Analysis of SCAMP1 expression in GC tissues and associated clinicopathological factors

Characteristics	SCAMP1		*P* value
	Low	High	
Age, years			0.207
<65	30	51	
≥65	12	34	
Gender			0.926
Male	29	58	
Female	13	27	
Size, cm			0.028*
≤4cm	19	22	
>4cm	23	63	
Tumor location			0.298
Upper third	6	15	
Middle third	5	9	
Lower third	26	40	
More than 2/3 stomach	5	21	
Tumor stage (Depth of invasion)			0.269
T1-T2	1	7	
T3-T4	41	78	
Lymph node metastasis			0.015*
Negative	15	14	
Positive	27	71	
TNM stage			0.266
I	1	3	
II	14	17	
III	27	65	
Lauren type			0.583
Intestinal	9	22	
Diffuse	33	63	

*pTNM stage* is defined using the American Joint Committee on Cancer (AJCC) Staging System, 8th Edition. Asterisk stands for *P* value as follows: *, *P* < 0.05.

**Table 3:** Univariate and multivariate *Cox* regression analyses for overall survival of gastric cancer patients.

Predictor	Univariate Analysis		Multivariate Analysis	
	HR (95% CI)	*P*	HR (95% CI)	*P*
Age, years				
≥65 vs <65	1.356(0.903-2.036)	0.142		
Gender				
Female vs Male	1.376(0.910-2.081)	0.130		
Size, cm				
>4cm vs ≤4cm	1.629(1.054-2.516)	0.028*	1.409(0.908-2.185)	0.126
Tumor location				
Middle 1/3 vs Upper 1/3	1.336(0.635-2.812)	0.445		
Lower 1/3 vs Upper 1/3	0.859(0.479-1.539)	0.609		
More than 2/3 stomach vs Upper 1/3	1.097(0.563-2.137)	0.785		
Tumor stage (depth of invasion)				
T3-T4 vs T1-T2	0.791(0.366-1.711)	0.552		
Lymph node metastasis				
Positive vs Negative	2.228(1.332-3.728)	0.002**	1.835(1.082-3.112)	0.024*
TNM stage				
II vs I	0.815(0.240-2.774)	0.744		
III vs I	1.833(0.577-5.824)	0.304		
Lauren type				
Diffuse vs Intestinal	1.004(0.623-1.617)	0.988		
Expression of SCAMP1				
High vs Low	1.919(1.219-3.021)	0.005**	1.638(1.033-2.597)	0.036*

HR, Hazard Ratio. *pTNM stage* is defined using the American Joint Committee on Cancer (AJCC) Staging System, 8th Edition. Asterisks stand for *P* values as follows: *, *P* < 0.05; **; *P* < 0.01.
